# Exploring the role of calf circumference as a predisposing factor for intra-hospital delirium: investigating potential gender differences: revealing potential gender variances

**DOI:** 10.1186/s12877-024-05334-1

**Published:** 2024-09-05

**Authors:** Chiara Ceolin, Mario Virgilio Papa, Cristina Simonato, Sara Cazzavillan, Margherita Vergadoro, Giulia Salerno Trapella, Riccardo Sermasi, Marina De Rui, Marianna Noale, Bruno Micael Zanforlini, Chiara Curreri, Anna Bertocco, Maria Devita, Giuseppe Sergi, Alessandra Coin

**Affiliations:** 1 Department of Medicine (DIMED) Geriatrics Division, University of Padua, Via Giustiniani 2, Padua, 35128 Italy; 2 Department of Neurobiology, Care Sciences and Society, Aging Research Center, Karolinska Institutet and Stockholm University, Stockholm, Sweden; 3 Department of Medicine (DIMED), Department of Women’s and Children’s Health, University of Padua, Padua, Italy; 4 Neuroscience Institute, National Research Council, Padua, Italy; 5 Department of General Psychology (DPG), University of Padua, Padua, Italy

**Keywords:** Delirium, Older adults, Calf circumference, Sarcopenia, Nutritional status

## Abstract

**Background:**

Malnutrition and sarcopenia significantly increase the risk of intra-hospital delirium, particularly among older adults. Given the potential correlation between calf circumference (CC) and these conditions, CC emerges as a promising predisposing factor for delirium. This study aims to investigate the independent association between delirium and anthropometric parameters, focusing on evaluating CC’s predictive capacity for intra-hospital delirium risk. Additionally, it aims to compare CC’s predictive performance with the widely used Mini Nutritional Assessment (MNA), while also considering potential gender disparities.

**Methods:**

This is a retrospective study which enrolled patients aged ≥ 65 years from September 2021 to March 2022 at the Padova Hospital (Italy). Physical characteristics, intra-hospital delirium incidence, and body composition were assessed. Sarcopenia was diagnosed using the 2019 European Consensus criteria.

**Results:**

Among 207 subjects, delirium affected 19% of patients. CC showed a significant association with intra-hospital delirium among the analyzed anthropometric parameters. ROC curves indicated that CC’s predictive capacity for delirium onset was comparable to MNA (*p* = 0.98), particularly in women. In a multivariable logistic regression model, female gender and higher cognitive and CC scores emerged as protective factors against delirium onset, with each unit increase in CC associated with a 24% reduction in the odds of delirium. Conversely, sarcopenia did not significantly influence delirium onset.

**Conclusions:**

CC shows promise as a predisposing factor for intra-hospital delirium, similar to MNA, albeit with significant gender differences. CC could serve as a valuable tool for assessing delirium risk among female patients. Further validation of these findings is necessary through larger-scale studies.

**Supplementary Information:**

The online version contains supplementary material available at 10.1186/s12877-024-05334-1.

## Background

Delirium is a geriatric syndrome characterized by acute and severe neuropsychiatric disturbances resulting in attention and cognitive impairments over a brief timeframe [14]. Its incidence spans between 8 and 17% among older patients in emergency departments, escalating to 40% among residents of nursing homes [2]. In the United States, it impacts over 2.6 million older adults each year, rendering it the most prevalent complication among this population [2]. Delirium is associated with significant clinical consequences such as increased frailty, mortality rates, re-hospitalizations, and institutionalizations among older adults, as well as substantial worldwide economic burden [14]. Hence, novel interventions targeting delirium prevention and mitigation of associated adverse outcomes and costs are imperative. Recognized assessment scales such as the Confusion Assessment Method (CAM) and the 4 ‘A’s Test (4AT) facilitate swift and efficacious diagnosis of delirium in clinical settings [27]. However, in hospital settings, the use of such assessment scales can often be challenging. Considering that among the main factors contributing to delirium incidence there are suboptimal nutritional status and dehydration, affecting both cognitively impaired and neurologically intact patients, it is reasonable to consider that indicators of nutritional status could be used as predictors of delirium onset during hospitalization [24]. In fact, there is evidence that scores on the Mini Nutritional Assessment (MNA), one of the most widely used malnutrition screening tools in the old population, correlates with scores on the 4AT [8]. Furthermore, malnutrition shares a complex relationship with muscle health, suggesting a possible link between lean mass and predisposition to delirium [3, 24]. An indicator that reflects both nutritional status and muscle health could be valuable for identifying patients at risk of delirium. According to the latest guidelines, the best methods to assess muscle mass are through instrumental examinations [28], however, due to the challenges in accessing these instrumental resources—especially for patients who are frail, difficult to transport, or uncooperative—it is crucial to have alternative methods for assessing body composition [5]. Our hypothesis is that calf circumference (CC) could serve as a reasonable alternative in these situations because it is considered a practical and feasible predictor of both nutritional and muscle status in older adults [5]. The purpose of this study is to estimate the risk of intra-hospital delirium incidence based on body composition measurements, considering also potential gender disparities. In particular, we aim to elucidate (1) the association between delirium and anthropometric parameters, with emphasis on CC; (2) the predictive capabilities of CC in discerning the risk of intra-hospital delirium in both genders, considering known variations in body composition, comparing it to the MNA; (3) whether CC can be considered an independent risk factor for delirium. In summary, our study not only addresses a critical gap in current literature but also pioneers a novel approach towards understanding and predicting intra-hospital delirium risk. By harnessing anthropometric indices, particularly CC, we aim to provide clinicians with a pragmatic tool for identifying and intervening in older adult individuals at heightened risk of delirium onset.

## Materials and methods

### Study population

This retrospective study involved a consecutive series of Caucasian patients recruited at the Geriatric Unit of the Azienda Ospedale Università Padova (Italy) between September 2021 and March 2022. The inclusion and exclusion criteria, as well as the participants’ characteristics, were detailed in a previous publication [5]. In summary, we included individuals over 60 years of age who had been hospitalized within the past 12 to 24 h. Conversely, those with fever, severe dehydration, or heart failure associated with significant body edema were excluded from the study. The study received the approval of the local Ethics Committee (protocol number 16412/AO/23). All patients gave oral and written informed consent to participate into the study.

### Data collection

In addition to the previously described information [5], data were also collected on:

#### Delirium assessment

The presence of delirium was investigated retrospectively, through a detailed analysis of the entire hospital stay using the patient’s medical records. To enhance sensitivity in detecting delirium within the study sample, we adopted an inclusive approach considering all potential keywords associated with delirium, as outlined in previous methodologies [19, 22], aligning with the Diagnostic and Statistical Manual of Mental Disorders (DSM-5) definition of delirium [12] by incorporating clinically identifiable features such as confusion, disorientation, altered mental status, delirium, agitation, inappropriate behavior, mental status change, inattention, hallucinations, lethargy. Patients were classified as delirious if they exhibited at least one of the following symptoms during hospitalization: reduced motor activity, lethargy, decreased responsiveness, delirium, confusion, and/or insomnia/restlessness. Subsequently, a binary variable indicating the presence or absence of delirium was established as the outcome variable.

#### Multidimensional geriatric assessment

As previously described [5], comorbidities (Cumulative Illness Rating Scale-CIRS), patients’ functional autonomy (Activities of Daily Living-ADL and Instrumental Activities of Daily Living), nutritional status (Mini Nutritional Assessment-MNA), and cognitive performance were assessed at admission. Moreover, through the collection of Exton Smith scale (ESS) for pressure sore risk, total number of patient’s medication and cohabitation status, the Multidimensional Prognostic Index (MPI) was calculated [20].

### Statistical analysis

The sample characteristics are presented as means ± standard deviation for normally distributed continuous variables, and as medians (interquartile range) for non-normally distributed continuous variables. The normality of these variables was checked using the Shapiro-Wilk test. Categorical variables are shown as counts and percentages. The characteristics of the participants were compared based on the presence of delirium using the Wilcoxon rank sum test or Fisher exact test, depending on the variable type. Associations between delirium and other variables were evaluated using Pearson, Spearman, or point biserial correlation tests, as appropriate, and visualized through heatmap analysis, with separate analyses by gender. Receiver Operating Characteristic (ROC) curves assessed the predictive ability of CC and the MNA score for intra-hospital delirium, with separate gender analyses. Logistic regression was used to examine the relationship between delirium and various covariates. Initially, univariate regression models evaluated the association of patient characteristics (age, gender, Mini Menta State Examination-MMSE at admission, MNA, Activities of Daily Living-ADL scores, number of comorbidities, and calf circumference) with incident delirium during hospitalization. Variables with a p-value < 0.20 were included in the multivariable model. Potential interactions between gender and other covariates were explored. Statistical analyses were performed using R software (version 4.1.1, R Foundation for Statistical Computing, Vienna, Austria). Logistic regression models were fitted with the ‘glm’ function from the ‘stats’ package. Odds Ratio plots were created with the ‘ggplot2’ package, while ROC curves were generated with the ‘pROC’ package. Comparisons of area under the curve (AUC) values were made using the ‘roc.test’ function from the ‘pROC’ package.

## Results

Table 1 shows the characteristics of the sample at admission according to the presence or absence of delirium during hospitalization; overall, 207 patients were enrolled for this study according to inclusion and exclusion criteria. The median age of participants with delirium was slightly higher compared to controls [86 (82;89) vs. 83 (78;88), *p* = 0.04]. Although not significant, there was a trend towards a lower presence of females among participants with delirium compared to controls (35% vs. 50.9%, *p* = 0.08). Some symptoms at admission, such as fever, dyspnea, and heart failure, showed no significant differences between groups. Functional assessments reveal significant differences between groups, with lower scores in MNA and MMSE in patients with delirium compared to controls [16.38 ± 4.50 vs. 18.27 ± 4.33, *p* = 0.02, and 17.30 (12.95;21.07) vs. 24.20 (19.70;27.85), *p* < 0.001, respectively]. Regarding body composition, CC was the only anthropometric parameter significantly different between the two groups (30.61 ± 4.45 vs. 32.38 ± 4.00, *p* = 0.02 among participants with delirium compared to controls, respectively). Finally, there were no significant differences in the prevalence of sarcopenia between groups. Regarding gender differences, we observed higher comorbidity and functional impairment in men with respect to women [CIRS-CI: 4.00 (2.25–5.75) vs. 3.00 (2.00–4.00), *p* = 0.04; ADL: 2.00 (1.00–6.00) vs. 1.00 (1.00–3.00), *p* = 0.02; IADL: 2.00 (1.00–5.00) vs. 1.00 (0.00-2.50), *p* = 0.005], while no differences were detected in age, MMSE scores, Body Mass Index-BMI, CC, and sarcopenia rates (data not shown).

**Table 1:** Sample characteristics at admission according to the presence or absence of delirium

Variables	All (*n* = 207)	Patients with delirium (*n* = 40)	Controls (*n* = 167)	*p*-value
Age [years]	84 (79;88)	86 (82;89)	83 (78;88)	***0.04***
Gender [F]	99 (47.4%)	14 (35%)	85 (50.9%)	0.08
*Symptoms and comorbidities*				
Fever	74 (36.6%)	16 (40%)	57 (35.7%)	0.71
Dyspnea	75 (35.1%)	10 (25%)	59 (36.9%)	0.19
Abdominal pain	12 (5.9%)	3 (7.5%)	9 (5.5%)	0.71
Infections/sepsis	85 (41.5%)	14 (35%)	69 (42.3%)	0.47
Heart failure	42 (20.3%)	4 (10%)	37 (22.2%)	0.38
Stroke	6 (2.9%)	1 (2.5%)	5 (3.1%)	1.00
Miscellanea	66 (32.2%)	21 (52.2%)	45 (27.6%)	***0.004***
CIRS-CI	3 (2;5)	5 (3;6)	3 (2;4)	***0.001***
O_2_ therapy [L/min]	0 (0;4)	0 (0;8)	0 (0;4)	0.96
N. drugs	6 (3;8)	6 (3;9)	6 (3;8)	0.53
*Functional evaluation*				
MNA	17.79 ± 4.42	16.38 ± 4.50	18.27 ± 4.33	***0.02***
MMSE	23.70 (18.15;27.70)	17.30 (12.95;21.07)	24.20 (19.70;27.85)	***< 0.001***
ADL	1 (1;4.25)	1 (0;2)	2 (1;6)	***0.003***
IADL	1 (0;4)	1 (0;1)	2 (1;5)	***< 0.001***
*Body composition parameters*				
BMI [Kg/m^2^]	27.00 ± 6.53	26.35 ± 4.86	27.21 ± 6.90	0.40
ASMMI [Kg/m^2^]	6.65 ± 1.14	6.53 ± 1.03	6.67 ± 1.16	0.62
FMI [Kg/m^2^]	10.64 (8.36;13.45)	9.87 (8.36;11.19)	11.03 (8.36;13.65)	0.16
MUAC [cm]	27 (24;29)	25 (24;28)	27 (24;30)	0.21
CC [cm]	31.98 ± 3.97	30.61 ± 4.45	32.38 ± 4.00	***0.02***
Max. handgrip strength [Kg_f_]	17.80 (11.85;25.35)	17.15 (11.98;24.60)	18 (11.80;26)	0.63
Sarcopenia [%]	41 (28.5%)	8 (40%)	33 (26.6%)	0.28

*Notes* Values are expressed as means ± standard deviation, medians (interquartile range) or counts (percentages), as appropriate

*Abbreviations* CIRS-CI = Cumulative Illness Rating Scale - Comorbidity Index; MNA = Mini Nutritional Assessment; MMSE = Mini Mental State Examination; ADL = Activities of Daily Living; IADL = Instrumental Activities of Daily Living; BMI = Body Mass Index; ASMMI = Appendicular Skeletal Muscle Mass Index; FMI = Fat Mass Index; CC = Calf Circumference; MUAC = Mid-Upper Arm Circumference

*P*-values < 0.05 are shown in bold

**Fig. 1:**
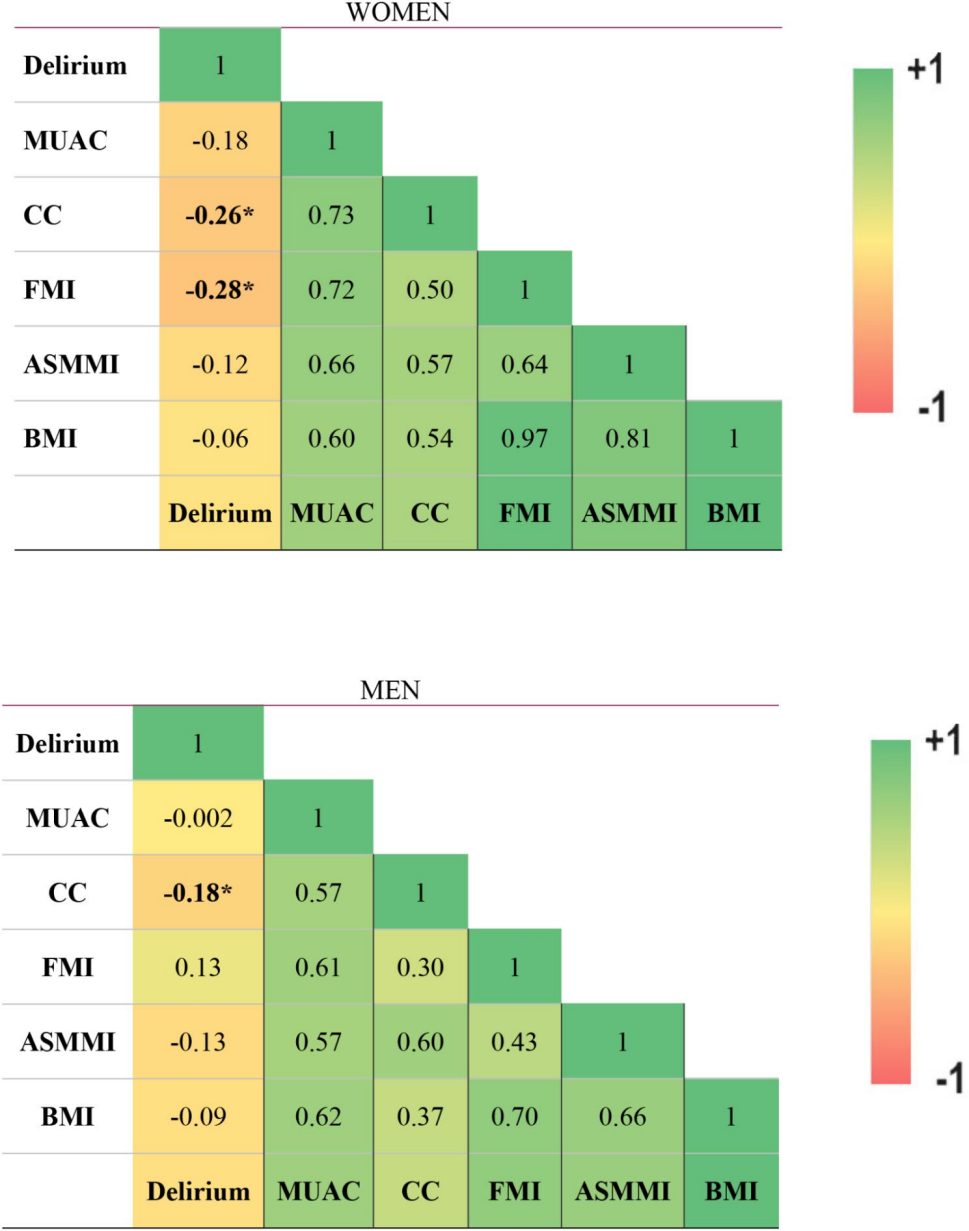
Simple correlations between anthropometric and body composition parameters and delirium, by gender. The Figure displays a heatmap representing, through color intensity, the correlations between delirium presence and anthropometric parameters. The only positive correlations are observed between delirium and calf circumference (CC) in both women and men (*p* < 0.05). In women, fat mass index (FMI) is also significantly associated with the presence of delirium. Abbreviations MUAC = Mid-Upper Arm Circumference; CC = Calf Circumference; BMI = Body Mass Index; ASMMI = Appendicular Skeletal Muscle Mass Index; FM = Fat Mass INdex; *p-values < 0.05 (only referred to delirium correlation)

In the analysis of correlations between anthropometric, body composition parameters and delirium, CC emerged as the sole parameter significantly associated with delirium (point biserial correlation 0.19, *p* = 0.03). This finding was further validated when analyses were conducted separately by gender (Fig. 1), revealing that in females, the Fat Mass Index-FMI was also significantly associated with delirium (*r*=-0.28, *p* = 0.02).

**Fig. 2:**
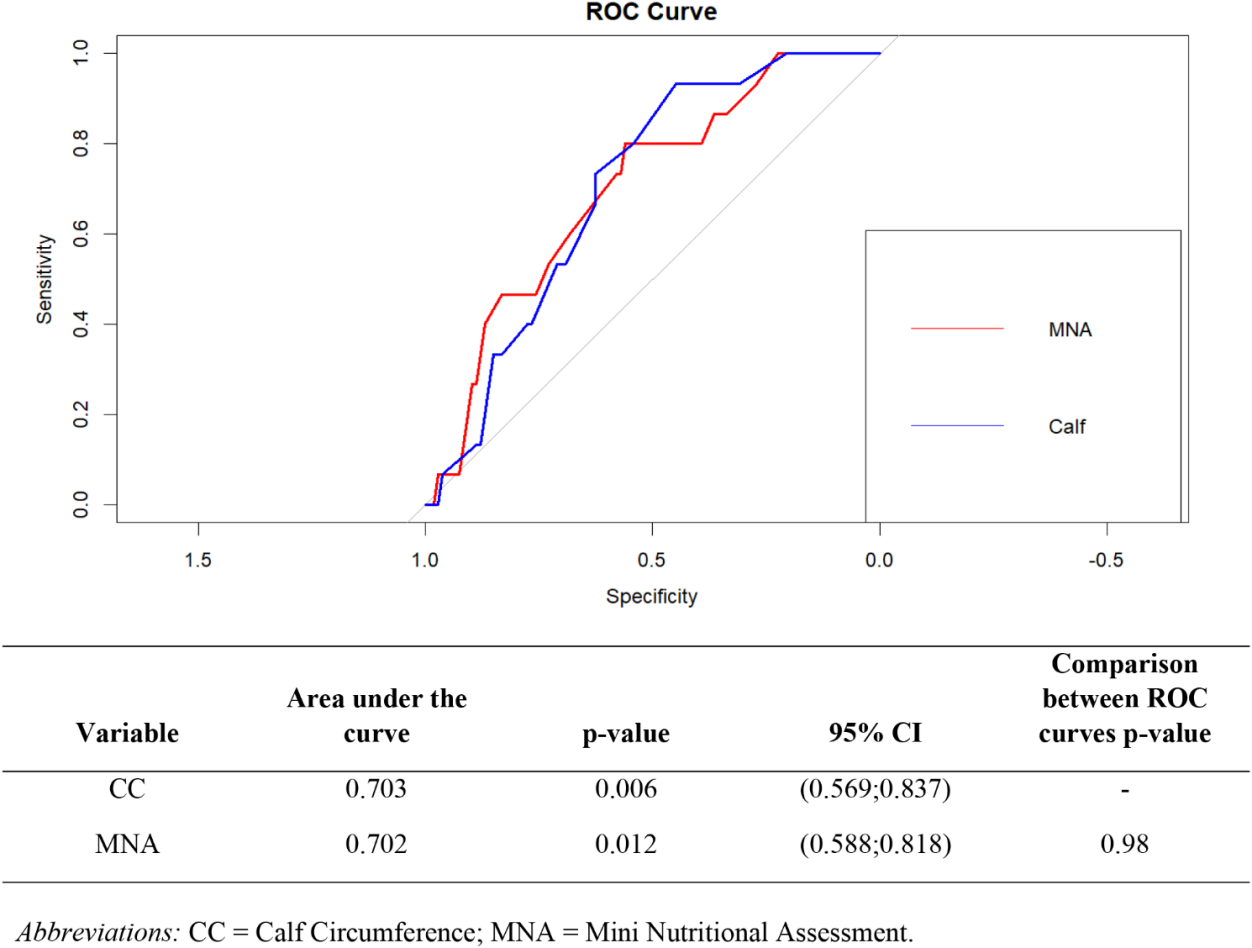
ROC curves for MNA and calf circumference in relation to delirium development. The ROC curve compares calf circumference (CC) and Mini Nutritional Assessment (MNA) in relation to delirium development. It illustrates the diagnostic performance of each measure by plotting the true positive rate (sensitivity) against the false positive rate (1-specificity) at various threshold levels. A higher area under the curve (AUC) indicates better overall accuracy in predicting delirium. This comparison helps to determine which measure is more effective in identifying patients at risk for delirium. *Abbreviations* CC = Calf Circumference; MNA = Mini Nutritional Assessment

The diagnostic accuracy of CC and MNA in predicting intrahospital delirium was assessed using their respective ROC curves. The results revealed that CC exhibited similar validity to MNA (AUC = 0.703, *p* = 0.006 and AUC = 0.702, *p* = 0.012, respectively, with a non-significant difference between the ROC curves) (Fig. 2). When analyzing predictive capacities stratified by gender, the findings observed in the overall sample were notably confirmed among females. This held true even when considering the influence of FMI. Specifically, CC demonstrated predictive capability comparable to both MNA and FMI (refer to Supplementary Fig. 1).

Table 2 presents the results of logistic regression analysis evaluating the association between patients’ characteristics and the likelihood of intrahospital delirium. Among the factors examined, female gender was associated with significantly lower odds of delirium (Odds Ratio (OR) = 0.10, *p* = 0.008, 95% Confidence Interval (CI) 0.01–0.46). Age, comorbidity burden (as measured by CIRS-CI), and the presence of sarcopenia did not show significant associations with delirium incidence. However, higher scores on the MMSE were significantly associated with decreased odds of delirium (OR = 0.83, *p* < 0.001, 95% CI 0.74–0.92). Similarly, each unit increase in CC was associated with a 24% reduction in the odds of delirium (OR = 0.76, *p* = 0.03, 95% CI 0.57–0.95]). The protective role of CC was confirmed also after adjustment for MPI values (please see Supplementary Table 1).

**Table 2:** Logistic regression of covariate-adjusted risk of intra-hospital delirium

Variable	OR	*p*-value	95 CI%	
			Lower limit	Upper limit
*Gender F*	0.10	***0.008***	0.01	0.46
*Age*	0.93	0.16	0.84	1.03
*CIRS-CI*	1.79	0.05	0.99	3.18
*Presence of Sarcopenia*	2.58	0.30	0.45	5.70
*MMSE*	0.84	***0.02***	0.79	0.97
*CC*	0.74	***0.04***	0.55	0.99

*Abbreviations* F: Females; CIRS-CI = Cumulative Illness Rating Scale - Comorbidity Index; MMSE = Mini Mental State Examination; CC = Calf circumference; OR = Odds Ratio; CI = Confidence Interval;. *P*-values < 0.05 are reported in bold

## Discussion

This is the first comprehensive assessment of gender disparities in delirium risk prediction, encompassing anthropometric, nutritional, and muscular indicators. Despite being aware of the complexity of delirium etiology, involving multifactorial contributors such as neuroinflammation, metabolic disturbances, and neurotransmitter dysregulation, calf circumference could represent a helpful tool. It has indeed been found to be significantly associated with the onset of in-hospital delirium, even after adjusting for other critical risk factors, including baseline cognitive level, comorbidities, and medication use, albeit with observed gender differences.

Consistent with existing literature [29], our study observed a delirium prevalence of 19% within the total sample. Moreover, we observed that delirium-affected patients exhibited advanced age, heightened comorbidity burden, compromised nutritional status, and diminished functional capacities, aligning with established associations linking delirium with age and chronic illnesses [13]. Among the various organic factors implicated in delirium onset, malnutrition, infections, surgery, and sarcopenia are recognized as key contributors to the development of intra-hospital delirium, independent of underlying dementia [6, 13]. However, estimating nutritional or muscular parameters in the old population is not always straightforward. Despite the array of assessment instruments at hand, like the MNA, anthropometry persists as a pragmatic, economical, and non-intrusive means of acquiring dependable nutritional indicators, particularly in cases where the patient is fragile, challenging to move, or uncooperative [5]. CC is regarded by the World Health Organization (WHO) as the most sensitive anthropometric indicator of muscle mass due to the fact that more than half of the body’s muscle mass is contained within the lower limbs [10]. Additionally, the leg area typically contains less adipose tissue, meaning that its circumference is predominantly influenced by muscle mass, rendering CC the most representative anthropometric measurement of body muscle mass [15]. Therefore, CC could serve as a valuable predictor of both nutritional and muscle status in the old population [4, 21, 23]. Given these premises, an association between CC and intra-hospital delirium would be hypothesised. While several studies have explored the link between sarcopenia and delirium, to our knowledge, only Zucchelli et al. [33] have examined the potential association between CC and delirium. In their study involving 1,675 hospitalized older adults, they identified CC as a useful indicator for intra-hospital delirium risk, particularly among individuals without dementia. However, their study lacked parameters for lean or fat mass comparison in evaluating the association between delirium and CC. Our study’s novelty lies in its comprehensive assessment of anthropometric parameters, encompassing measurements of both lean and fat mass. Furthermore, our study aimed to delve into gender differences in assessing these parameters, recognizing the known variations in body composition between males and females [7]. Interestingly, among the anthropometric parameters evaluated, CC emerged as the sole factor associated with delirium. From the limited studies available in the literature, it emerges that low BMI levels are a risk factor for the onset of delirium, both in medical and surgical wards [11, 16]. However, certain measures such as BMI exhibit diminished accuracy in older adults due to age-related changes in body composition and challenges in obtaining height and weight measurements in older individuals [18, 25, 32]. In instances where BMI assessment is unfeasible, CC serves as an excellent alternative. Additionally, we found that CC diagnostic accuracy in predicting intra-hospital delirium was comparable to that of MNA. To our knowledge, no studies have directly compared CC and MNA score indices in predicting delirium, as CC is typically only incorporated into more complex tools and used solely in predicting malnutrition [26]. Nevertheless, these findings underscore the dual role of CC as a predictor of both nutritional and muscular status. Intriguingly, our study revealed that its predictive capacity was more pronounced in women than in men. This is likely related to physiological differences in body composition and the impact of sarcopenia between genders. Women generally have a higher percentage of body fat and lower muscle mass compared to men, which can influence how muscle mass is assessed using calf circumference [33]. For women, a smaller calf circumference may more accurately reflect lower skeletal muscle mass. Furthermore, previous research indicates that a lower calf circumference is a stronger independent risk factor for poor physical performance and related conditions in women than in men [31]. This suggests that women may be more sensitive to changes in muscle mass, making calf circumference a more reliable indicator of their health status. These observations are supported by the stronger correlations observed between body composition indicators in women compared to men. Despite not assessing participants’ levels of physical activity, in our study, the higher comorbidity and functional impairment among men may partly account for these results.

Finally, in our logistic regression model higher MMSE scores were found to be protective against the onset of delirium, consistent with the well-established association between delirium onset and cognitive impairment [9]. Our findings regarding gender align with previous research, highlighting males as being at higher risk of delirium [17, 30]. These findings are partly supported by hormonal and metabolic differences: elevated levels of estradiol are associated with an increased incidence of delirium [1]. Therefore, considering the physiological decline in sex hormones during menopause in women, it is plausible that the risk of delirium decreases.

Our study presents several significant limitations that deserve attention. Firstly, being a retrospective study, there is a likelihood of underdiagnosis of hypoactive delirium, a form of delirium that is often more difficult to identify compared to other variants. This aspect may influence our overall understanding of the prevalence of this condition in hospitalized patients. Additionally, the relatively small sample size limits the generalizability of the results. With a limited number of cases, it is challenging to make definitive claims that can be applied to a broader population. Finally, another significant constraint was the inability to determine the prevalence of patients using anticholinergic and sedative medications, which can have a considerable impact on the diagnosis and management of delirium. Despite these limitations, it is important to emphasize that the prevalence of delirium observed in our sample, estimated at around 20%, is consistent with findings reported in other studies on hospitalized patients. This suggests that, although there are critical issues in our approach, the results regarding delirium prevalence are in line with existing literature. Moreover, the comprehensive approach used enabled us to correlate CC values with clinically relevant variables. Our study was the first to explore the potential relationship between gender, anthropometry, and the onset of delirium in an aging patient cohort. Moving forward, future research endeavors should prioritize the inclusion of larger and more diverse samples to validate and expand upon our findings.

In conclusion, our findings underscore the significance of CC as a dual indicator of nutritional and muscular status among older patients, particularly in predisposing to intra-hospital delirium. Notably, CC demonstrated a comparable predictive capacity to MNA in identifying patients at risk of delirium onset, with each unit increase in CC associated with a 24% reduction in the odds of delirium. Furthermore, our study revealed that the predictive capability of this anthropometric parameter was more prominent in women compared to men, indicating potential gender disparities in delirium risk assessment. While further research is warranted to elucidate the underlying mechanisms driving these gender-specific differences, our study provides valuable insights into the utility of anthropometric measures, such as CC, in identifying patients at risk of delirium onset. Moreover, our study serves as an initial step in exploring the relationship between gender, anthropometry, and delirium onset in older patients, laying the foundation for future investigations in this area.

## Electronic supplementary material

Below is the link to the electronic supplementary material.

Supplementary Material 1

## Data Availability

The datasets used and analyzed during the current study are available from the corresponding author on reasonable request in the manuscript, whereas on the system you have provided.

## Abbreviations

ADL: Activities of daily living
ASMMI: Appendicular skeletal muscle mass index
BMI: Body mass index
CC: Calf circumference
CIRS: Cumulative illness rating scale
FMI: Fat mass index
IADL: Instrumental activities of daily living
MNA: Mini nutritional assessment
MMSE: Mini-mental state examination
MUAC: Mid-upper arm circumference
