# Supplementary material for: Exploring the role of calf circumference as a predisposing factor for intra-hospital delirium: investigating potential gender differences: revealing potential gender variances
Source: BMC Geriatr. 2024 Sep 5;24:739. doi: 10.1186/s12877-024-05334-1 (PMC11378412; doi:10.1186/s12877-024-05334-1)
Supplement: Supplementary file 1 — Supplementary Material 1 [file 12877_2024_5334_MOESM1_ESM.docx]

**Supplementary Figure 1. ROC Curves for MNA and Calf Circumference in Relation to Hyperkinetic Delirium Development, by gender.**

*The ROC curve compares calf circumference (CC) and Mini Nutritional Assessment (MNA) in relation to delirium development. It illustrates the diagnostic performance of each measure by plotting the true positive rate (sensitivity) against the false positive rate (1-specificity) at various threshold levels. A higher area under the curve (AUC) indicates better overall accuracy in predicting delirium. This comparison helps to determine which measure is more effective in identifying patients at risk for delirium.*


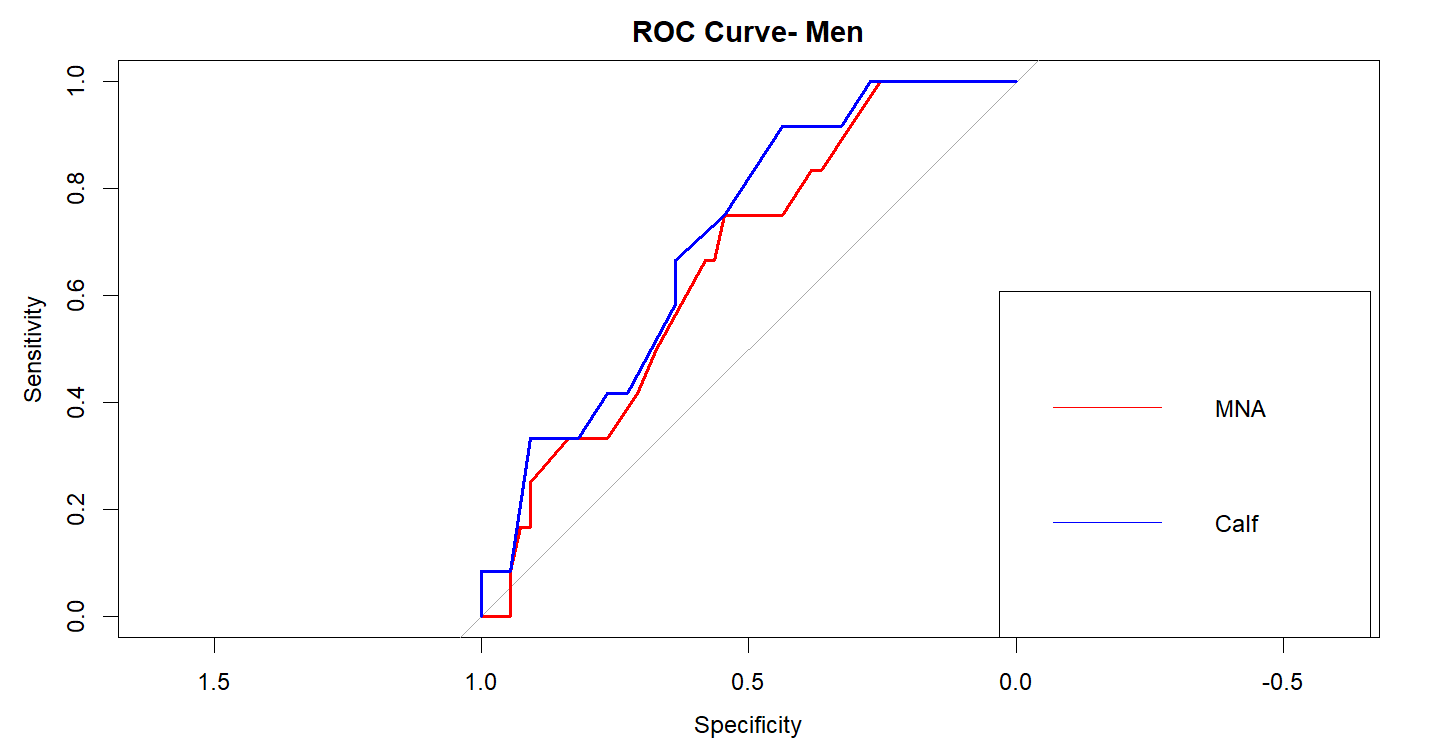


| **Variable** | **Area under the curve** | **p-value** | **95% CI** | **Comparison between ROC curves p-value** |
| --- | --- | --- | --- | --- |
| CC | 0.704 | 0.15 | (0.557;0.850) | **-** |
| MNA | 0.660 | 0.33 | (0.505;0.816) | 0.62 |


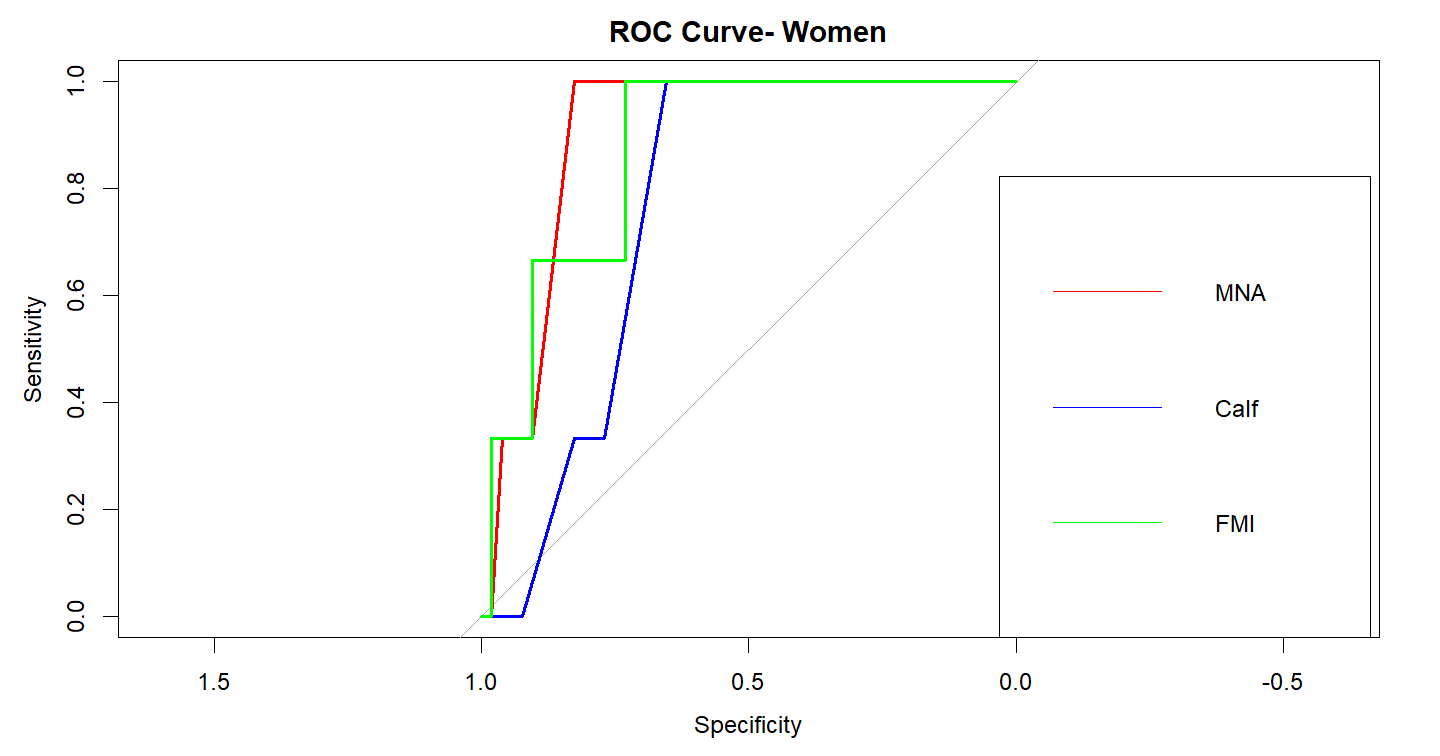


| **Variable** | **Area under the curve** | **p-value** | **95% CI** | **Comparison between ROC curves p-value** |
| --- | --- | --- | --- | --- |
| CC | 0.766 | 0.04 | (0.617;0.915) | **-** |
| MNA | 0.901 | <0.001 | (0.802;0.999) | 0.19 |
| FMI | 0.872 | 0.003 | (0.713;0.999) | 0.45 |

*Abbreviations:* CC = Calf Circumference; MNA = Mini Nutritional Assessment; FMI = Fat Mass Index.

**Supplementary Table 1. Logistic regression of covariate-adjusted risk of intra-hospital delirium.**

| ***Variable*** | ***OR*** | ***p-value*** | ***95 CI%*** | |
| --- | --- | --- | --- | --- |
|  |  |  | **Lower limit** | **Upper limit** |
| ***Gender F*** | 0.13 | ***0.01*** | 0.03 | 0.46 |
| ***Age*** | 0.96 | 0.46 | 0.87 | 1.06 |
| ***CIRS-CI*** | 1.35 | 0.09 | 0.96 | 1.97 |
| ***Presence of Sarcopenia*** | 0.45 | 0.37 | 0.08 | 2.25 |
| ***MMSE*** | 0.83 | ***<0.001*** | 0.74 | 0.92 |
| ***MPI*** | 0.28 | 0.35 | 0.03 | 2.51 |
| ***CC*** | 0.76 | ***0.03*** | 0.57 | 0.95 |

*Abbreviations:* F: Females; CIRS-CI= Cumulative Illness Rating Scale - Comorbidity Index; MMSE = Mini Mental State Examination; MPI = Multidimensional Prognostic Index; CC = Calf circumference; OR = Odds Ratio; CI = Confidence Interval. P-values<0.05 are reported in bold.
